# Weathering of a Roman Mosaic—A Biological and Quantitative Study on *In Vitro* Colonization of Calcareous *Tesserae* by Phototrophic Microorganisms

**DOI:** 10.1371/journal.pone.0164487

**Published:** 2016-10-26

**Authors:** Addolorata Marasco, Simona Nocerino, Gabriele Pinto, Antonino Pollio, Giorgio Trojsi, Antonino De Natale

**Affiliations:** 1 Department of Mathematics and Applications, University of Naples Federico II, Complesso Universitario di Monte Sant’Angelo, Via Cintia, 80126 Napoli, Italy; 2 Via C. Ravizza, 20149 Milan, Italy; 3 Department of Biology, University of Naples Federico II, Complesso Universitario di Monte Sant’Angelo, Via Cintia, 80126 Napoli, Italy; 4 Università degli Studi Suor Orsola Benincasa, Via Santa Caterina da Siena, 80135 Napoli, Italy; University of New South Wales, AUSTRALIA

## Abstract

The potential impact of cyanobacteria and microalgae on the weathering of calcareous *tesserae* from a Roman mosaic of the II Century CE has been followed through *in vitro* experiments. Laboratory tests were carried out by inoculating mosaic tiles with single strains of Cyanobacteria or Chlorophyta to evaluate the roles of pioneer phototrophic microrganism on the resulting architecture of biofilms. The interaction between *tesserae* and strains was assessed at the whole substratum and micrometer scales, by image analysis and Confocal Laser Scanning (CLS) microscopy, respectively. The biofilm surface coverage on each *tessera* varied from 19% (*Fischerella ambigua*) to 97% (*Microcoleus autumnalis*). Cyanobacteria showed a better growth on calcareous *tesserae*, whereas the only green alga attaining a superficial coverage higher than 50% was *Coelastrella rubescens*. CLS microscopy evidenced two different types of spatial arrangement of the phototrophic organisms on the *tesserae*, that were defined as compact or porous, respectively. In the first one was measured a reduced number of empty spaces between cells or filaments, whereas in the second type, a reticulate texture allowed the presence of numerous empty volumes. The colonization processes observed are an intrinsic characteristic of each strain. We have proposed a *colonization index* *I*_*C*_ as a sensible tool to describe, in a quantitative way, the pioneering attitude of each photosynthetic microorganism to colonize lithic substrates under laboratory conditions.

## Introduction

Mosaics are one of the most typical works of art in Roman architecture. Built with small squares called *tesserae* and made with various materials (marble, glass paste and stones), they were largely used to decorate the floors of private houses and public buildings, beginning in the 3^rd^ century BC [1]. Their close contact with the ground and direct exposure to sun light and changing climatic conditions represent a severe challenge to their conservation. Mosaics are very frequently exposed to biofilm attachment, as irradiance and relative humidity values frequently allow the settlement of microorganisms. Biological development, in addition to climatic conditions, is also influenced by the intrinsic characteristics of the substrate, defined as bioreceptivity [2]. These characteristics relate to the physical properties (e.g., porosity and roughness properties) and chemical properties (e.g., the chemical composition) of the substrate. The contraction and swelling of microbial biofilms, the penetration of filaments and salt bindings due to the production of extracellular polymeric substances (EPS) can greatly weaken stone substrata [3]. Moreover, due to the growth of biofilm between the single *tesserae*, the stability of the entire mosaic can be seriously compromised over the course of years, leading to the detachment of parts of the structure.

**Fig 1:**
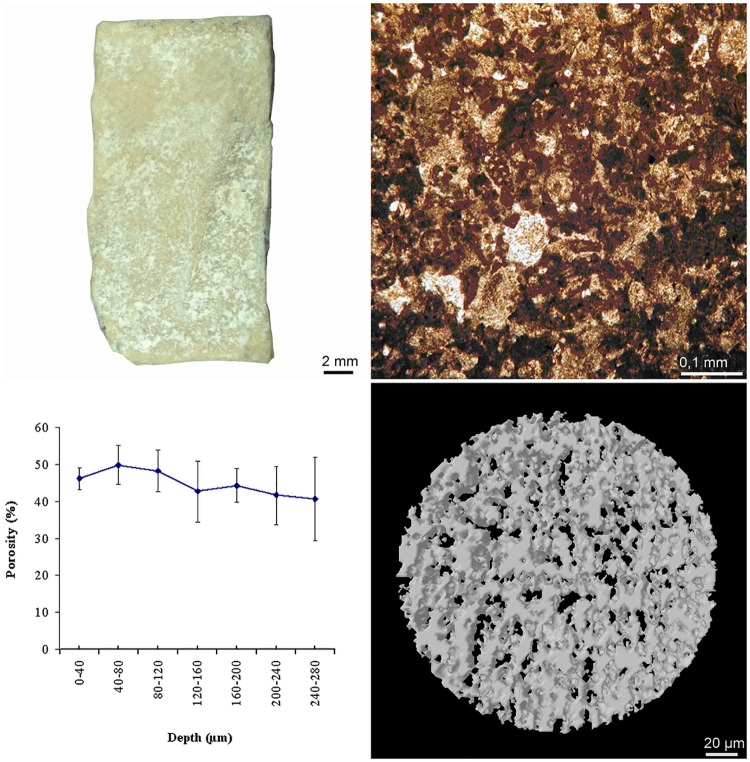
(left top panel) a mosaic *tessera* from the thermal baths of via Terracina; (right top panel) image of the polarized light from a *tessera* (100X; N+); (left bottom panel) porosity distribution between 0-280 μm in depth; and (right bottom panel) 3D reconstruction of the superficial *tessera* layers (280 μm in depth).

**Fig 2:**
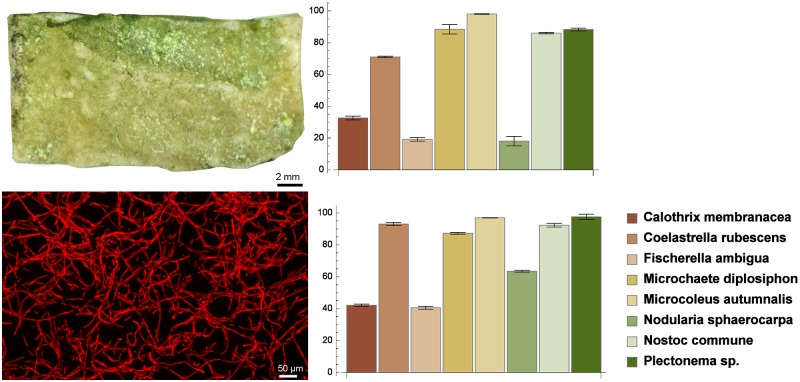
(left panels) *Calothrix membranacea* on *tessera* (top) and MIP of *Calothrix membranacea* (bottom); (right panels) substratum coverage on *tesserae* (top) and on MIPs (bottom) as a percentage on the available area (mean ± sd).

It is well known that phototrophic microorganisms play a key role in biofilm establishment. The intensive growth of phtototrophic microganisms and lichens on and between the *tesserae* of Roman mosaics is considered one of the most important decay factors [4], with field xerophytic conditions supporting the establishment of lichens, whereas the high humidity promotes cyanobacterial and algal growth [5, 6]. There is increasing evidence for the role that each single species of microorganisms can have in the first steps of biofilm formation. Curtis and Sloan [7] reported that structure and composition of a biofilm are determined by the pioneer species, which first colonize a substrate. Urzì et al. [8] also stressed that the variability within the same species can be so high, it could cause different types of colonization, even in microbial communities growing in close vicinity. For this reason, we planned a laboratory study on the bioreceptivity of original *tesserae* from a Roman Mosaic (Fig 1, left top panel), with the aim of comparing the ability of attachment and growth on the substratum of Cyanobacteria and Chlorophyta strains selected between those most frequently present on lithic monuments [9]–[19] (see Fig 2). The *tesserae* were previously characterized for their mineralogical and petrographic features and then inoculated with a single phototrophic microorganism. The three-dimensional structure of the microbial population on the substrate was studied using both image analysis and CLS microscopy. An additional purpose of this paper was to analyze their distribution pattern on the substrate, thus providing quantitative tools to describe the complexity of their architecture. Finally, an index was proposed to describe the colonization ability, according to either the compact or porous growth structure of each strain.

## Materials and Methods

### Mineralogical and Petrographic analysis

The Roman Thermal Baths of via Terracina in Naples, Italy, is an archaeological area with several levels built in the 2^nd^ century BC. Placed at the halfway point between Puteoli and Neapolis, the thermal baths were a place to rest for a short time (geographical coordinates: 40° 49’ 49.43” N 14° 11’ 20.36” E). The baths are constituted by two principal areas (*frigidarium* and *apodycterium*) and several environments (*corridor* and *tabernae*), which were later added to the early structure. All of the floors of the thermal baths were originally covered by mosaics, but now they are partially or completely destroyed. This study was carried out in the year 2009 with the permission of the “Soprintendenza Archeologica di Napoli e Pompei” (now this archaeological area is under the authority of “Soprintendenza Archeologica della Campania” [20]). Part of the *frigidarium* and *apodycterium* pavements does not receive direct sunlight for most of the year, and the light irradiance rarely exceeds 200 *μ*mol photon m^−2^ s^−1^. In these parts of the mosaic, the *tesserae* suffer from a prolonged water stagnation after rain, remaining submerged for days. Standing waters allow the proliferation of algal populations, that represents one of the major causes of biodeterioration of the floor.

For the petrographic analyses, small fragments of the two mosaic *tesserae* were ground and finely pulverized to improve the counting statistics. An X-ray diffraction (XRD) based irradiance of the sample, with a monochromatic beam produced by an appropriate generator, was performed with a Rigaku Miniflex 600 X-ray diffractometer with a cobalt tube operating at 30 KV and 15 mA, and wherein the sample of the molecular crystal is scanned over a region of 2-40° (2*θ*) per minute with a step size of 0.05° and a counting time of 3600 seconds. For the estimations of the mineral phases in the bulk rock, the following peak-height intensities for diagnostic reflections were used: calcite, 3.03 Å; quartz, 3.34 Å; mica, 10 Å; clay minerals, 4.43-4.48 Å; siderite, 2.79 Å; goethite, 4.18 Å; maghemite, 2.51 Å; pyrolusite, 3.11-3.16 Å; and hausmannite, 2.49 Å. The petrographic analyses of the samples were conducted on thin sections of *tesserae* under a Nikon Eclipse E400 Pol petrographic microscope using polarized light to clarify and complete the identification obtained using XRD. The analyses carried out with the aid of XRD and optical microscopy on thin sections (TS) were conducted in the diagnostic laboratory of the Suor Orsola Benincasa University of Naples. Before assessing the density and water adsorption coefficient, eight mosaic *tesserae* were held at a constant temperature (21°C) and relative humidity (51-55%) for two weeks. The density (mean ± sd) of the *tesserae* was obtained by evaluating the ratio weight/volume. The water absorption coefficient (WAC) of the *tesserae* (mean ± sd) was calculated according to Barberousse et al. [21].

### Roughness and Porosity

The roughness parameters were evaluated on each *tessera* with an ALPA^©^ RT-20 palmar rugosimeter, as described in the standard ISO 4287:1984 [22]. The measurements consisted of analyzing each *tessera* with 1600 sampling points (with a cut-off of 0.625 μm). All measurements were performed in triplicate, and data acquisition was conducted using the Measurement Studio Lite 1.0.3.96 software. The porosity of *tesserae* was measured using X-ray micro-computed tomography (μCT) with a Skyscan 1074 [23]. The samples were scanned at a voltage of 39 kV with a current intensity of 921 μA; the total duration for tomographic imaging was approximately 4 h (exposure 3540 ms). The measurements were performed in triplicate.

### Origin and culture of the strains

The experiments were conducted with algal strains from the ACUF culture collections (www. acuf.net) of the Department of Biology, University of Naples Federico II. The Chlorophyta strains included the following: *Chlorella vulgaris* Beij. (ACUF056), *Coccomyxa solorinae-saccatae* Chodat (ACUF198), *Coelastrella rubescens* (Vinatzer) Kaufnerová et Eliás (ACUF275), *Pseudococcomyxa simplex* (Mainx) Fott (ACUF047), *Scenedesmus communis* Hegewald (ACUF280), and *Stichococcus bacillaris* Nägeli (ACUF211). The Cyanobacteria included the following: *Aulosira terrestre* Subba-Raju (ACUF246), *Calothrix membranacea* Shmidle (ACUF114), *Fischerella ambigua* (Kützing ex Bornet et Flahault) Gomont (ACUF304), *Microchaete diplosiphon* Gomont ex Bornet et Flahault (ACUF300), *Microcoleus autumnalis* (Gomont) Strunecky, Komárek et Johansen (ACUF324), *Nodularia sphaerocarpa* Bornet et Flahault (ACUF033), *Nostoc commune* Vaucher ex Bornet et Flahault (ACUF299), *Plectonema* sp. (ACUF589), and *Scytonema mirabile* Bornet (ACUF590). The strains were cultured in Bold Basal Medium (BBM) [24] in 100 ml Erlenmeyer flasks. The flasks were put in a climatic chamber at 20 ± 2°C and illuminated by growth light Osram white fluorescent lights, with a 16:8 h light dark cycle, at an irradiance of 150 *μ*mol photon m^−2^ s^−1^. The irradiance was measured with a LI-COR Biosciences radiation (data logger LI-1400, quantum sensor LI-190).

### Accelerated colonization

To reproduce the peculiar situation of immersion suffered from part of the *tesserae* of the mosaic, previously sterilized *tesserae* (average dimensions 2.5cm × 1.2cm × 1.07cm (see Fig 1, left top panel) were placed in horizontal orientations in 100 ml Erlenmeyer flasks, each containing 50 ml of BBM medium. The flasks were previously inoculated with a single algal strain and, when the cultures were in the mid-exponential phase of growth at approximately 0.3 optical density (OD), a single *tessera* was aseptically added to each flask. We remark that the value of 0.3 OD was chosen on the basis of results from preliminary experiments, showing that inocula with lower cell concentrations led to an increase of variability of surface coverage of *tesserae* by each strain, and then to a reduced reproducibility of the tests. The strains were grown in the same culture conditions, as previously described, and the incubation time was set at 1 month, as in other accelerated biofouling tests [21, 25, 26]. At the end of the tests, the *tesserae* were aseptically picked from the flasks by gently tilting the flasks to remove the algae that were loosely attached. Then, *tesserae* were transferred to sealed glass chambers and kept in the same conditions of light and temperature, as previously described. Each chamber had a lower part filled with tap water, in which was placed a perforated ceramic grille that supported open Petri dishes containing the *tesserae*. Under these conditions, the relative humidity was maintained in the range of 98-100% throughout the experiments. After two weeks, each *tessera* was digitally photographed and subsequently observed with a CLS microscopy.

### Image analysis

Digital image analysis was applied to quantify the microbial growth on the stone samples after the incubation time. The samples were taken out of the cell chamber and placed on filter paper to remove excess water. The photographic recording of each *tessera* was performed in triplicate with a digital camera (Nikon D5100). Because the color of the *tesserae* (pale gray) did not mask the presence of the microbial population, conventional RGB color images obtained with the digital camera could be used to quantify the colonization area on each *tessera*. Using the open source software Gimp^®^ for manipulating images (http://www.gimp.org), the colonized areas of each photograph were measured. This software allowed us to analyze the portions of each image, characterize them by shape or color (with a range of shades), and finally evaluate the number of pixels selected in this way. Then, the empty and colonized areas on each *tessera* were identified by means of the shape and color, respectively. Finally, the ratio of the number of the selected pixels on the colonized and empty areas in each *tessera* determined the substratum coverage. Similarly, the maximum intensity projections (MIPs) constructed from the confocal Z-stacks were analyzed with Gimp^®^.

### CLS Observations

The microbial populations on the substrate were also analyzed with Zeiss LSM 700 (software Zen 2011) by capturing images at 10x that of the phototrophic microorganisms autofluorescence (excitation beams at 543 mm and 633 nm and emission at 590-800 nm). The *tesserae* used in the experiments were selected from those that have little or nothing interference with the microbial autofluorescence. For each *tessera*, three sampling points were used to build the Z-stacks (see S1 Table). The thickness of each stack varied between 5 and 6 μm. The acquisitions of the three Z-stack of *tesserae* with different thicknesses have been adapted to obtain the same number of slices. The open source image processing package Fiji ([27], and also http://www.fiji.sc) was used to evaluate the area and perimeter of all stacked CLS images, and to obtain 2D MIPs. The images have been previously converted to 8-bit and then resampled by using the tool *Threshold* [28]–[30]. *Analyze Particles* tool was used to determine the areas and perimeters of each slice belonging to Z-stacks [31]–[32]. In addition, the plugin Fractal Dimension, applying a box-counting algorithm, evaluated the *fractal dimensions* of each organism pattern (www.bonej.org/fractal).

### Mathematical and statistical analysis

#### Fractal dimension

We recall that the box-counting fractal dimension of a subset F of ℜ^*n*^ is [33]:

$$\begin{array}{c} D_{B}(F)=\lim_{\delta \to 0}\frac{\ln N_{\delta }(F)}{-\ln\delta }, \end{array}$$ (1)

where *N*_*δ*_(*F*) is the smallest number of cubes of side *δ* that cover *F*. The plugin *Fractal Dimension* estimated *D*_*B*_(*F*) as the slope of a regression line through the points (ln *N*_*δ*_(*F*), −ln *δ*) on decreasing the box size *δ*. Moreover, the plugin also supplied the coefficient of determination (*R*^2^) of this linear regression (see S2 Table).

#### Statistical description of data

The values of area, perimeter, and fractal dimension relative to the colonized areas obtained from the stacked CLS images (for each microbial population, we had three sets of *N*_*i*_ data, where *N*_*i*_ is the number of CLS images for *i*–th algal population, *i* = 1, …, 8 (see S1 Table, first column)) were summarized in the box and whisker plots (see S1–S3 Figs, upper panels). Moreover, these three sets of data were compared pairwise to test their statistical indistinguishability using the *Mann-Whitney U-tests* at a significance level of 0.05 (see S1 Text and S1–S3 Figs, bottom panels). A box and whisker plot was used to represent the roughness data of each *tessera*, then these three sets of 1600 data were compared pairwise to test their statistical indistinguishability. The substratum coverage (mean ± sd) of the microbial populations on mosaic *tesserae* and on the MIPs of confocal Z-stacks were plotted in a bar chart, and Pearson’s correlation was analyzed. All of the graphical representations and the statistical tests were performed using the software *Mathematica*^®^ (ver. 10.4 Wolfram).

#### Selected parameters to describe the microbial architecture

First, we defined *substratum coverage* as the fraction of area covered by the microbial biomass. We note that the substrate here was generally defined as the interface that supported microbial growth. Then, the substratum coverage of the microbial populations on mosaic *tesserae* (resp. MIP) were calculated as $A_{i}^{S}/a_{i}^{S}$, where $A_{i}^{S}$ is the microbial area on the *i*–th *tessera* (resp. MIP) and $a_{i}^{S}$ is the total area of the *tessera* (resp. MIP). Similarly, for the *i*–th population, the substratum coverage on each slice of the confocal Z-stacks was calculated as *A*_*ij*_/*a*_*i*_, where *A*_*ij*_ is the microbial area in the *j*–th slice and *a*_*i*_ is the area of the slice. For all of the CLS images relative to each microbial population, we determined the mean and standard deviation for the area, perimeter, and fractal dimension relative to the three sampling points (S4 and S5 Figs). We noted that instead of a single mean value±sd for the area, perimeter, and fractal dimension, we obtained three sets of Ni mean values±sd (*i* = 1, ..8). To compare and describe the microbial architectures, we considered the following dimensionless quantities:

- Substratum coverage
  $$\begin{array}{c} A_{ij}=\frac{A_{ij}}{a_{i}},\;j=1,...,N_{i} \end{array}$$ (2)
  where *A*_*ij*_ is the microbial area in the *j*–th slice, and *a*_*i*_ is the area of slice.
- The ratio between the characteristic linear measure of the microbial pattern and that of the slice
  $$\begin{array}{c} L_{ij}=\frac{A_{ij}/P_{ij}}{\sqrt{a_{i}}/4},\;j=1,...,N_{i}. \end{array}$$ (3)
- The normalized value of the fractal dimension
  $$\begin{array}{c} D_{ij}=\frac{D_{B}(F_{ij})}{2},\;j=1,...,N_{i} \end{array}$$ (4)
  where *D*_*B*_(*F*_*ij*_) is the box-counting fractal dimension of the *i*–th algal population into the *j*–th slice.

It is easy to verify that

$$\begin{array}{c} 0\leq A_{ij},\;L_{ij},\;D_{ij}\leq 1,\;\forall i,j. \end{array}$$

**Fig 3:**
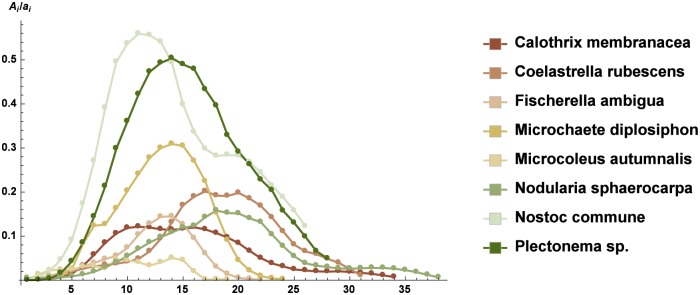
Substratum coverage of microbial populations on each slice of the Z-stacks (mean ± sd). On the *x*–axis are reported the numbered slices in the stacks of each strain from the bottom to the top of the biofilm (see S1 Table).

**Fig 4:**
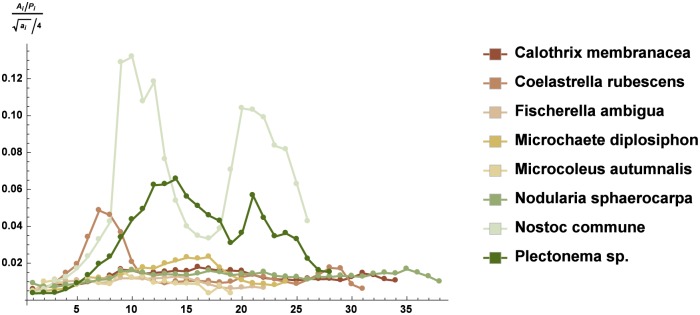
The ratio between the characteristic linear measure of the microbial pattern and that of the slice relative to the Z-stacks (mean ± sd). On the *x*–axis are reported the numbered slices in the stacks of each strain from the bottom to the top of the biofilm (see S1 Table).

**Fig 5:**
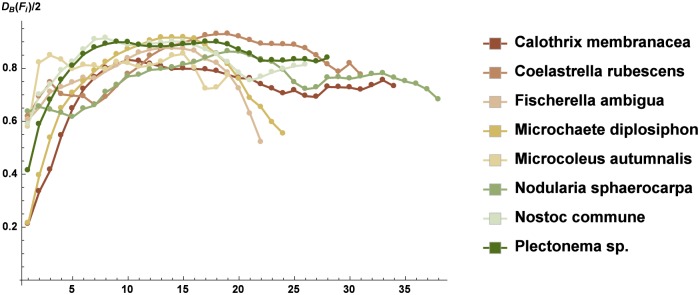
The normalized value of the fractal dimension of each slice of the Z-stacks (mean ± sd). On the *x*–axis are reported the numbered slices in the stacks of each strain from the bottom to the top of the biofilm (see S1 Table).

Figs 3–5 were drawn using the quantities in Eqs (2)–(4).

#### Statistical aggregation operators

We needed to aggregate, in a meaningful way, the *N*_*i*_ values in Eqs (2)–(4) for *i* = 1, … 8. Therefore, we proposed three different aggregation operators for each set of *N*_*i*_ values referring to the *i*–th population (see S3–S5 Tables).

- **Maximum operator**
  $$\begin{array}{c} S_{i}^{\max}=\max(S_{i1},\cdots ,S_{iN_{i}}),\;i=1,...8 \end{array}$$ (5)
  where $S_{ij}$ is one of the quantities in Eqs (2)–(4).
  This operator takes on the largest of any of the values $S_{ij}$, and is zero if and only if $S_{ij}$ is zero for all *j*. Then, $S_{i}^{\max}$ allows the use of information only from the maximum value of $S_{ij}$.
- **Average operator**
  $$\begin{array}{c} {\bar{S}}_{i}=\frac{1}{N_{i}}\sum_{j=1}^{N_{i}}S_{ij},\;i=1,...8. \end{array}$$ (6)
- **Weighted average operator**
  $$\begin{array}{c} S_{i}^{w}=\sum_{j=1}^{N_{i}}w_{ij}S_{ij},\;i=1,...8 \end{array}$$ (7)
  where *w*_*ij*_ is a suitable weight for *j*–th value $S_{ij}$, i.e.,
  $$\begin{array}{c} w_{ij}\geq 0,\;\sum_{j=1}^{N_{i}}w_{ij}=1. \end{array}$$
  Due to the distribution of the values $S_{ij}$ in any CLS image of *i*–th population (Figs 3–5 and S4 and S5 Figs), we proposed to use the Gaussian probability density function (PDF) to generate the weights *w*_*ij*_ as follows. We recall that the PDF of Gaussian distribution for a continuous random variable *x* is defined as
  $$\begin{array}{c} f(x)=\frac{1}{\sigma \sqrt{2\pi }}\exp(-\frac{{\left(j-\mu \right)}^{2}}{2\sigma }),\;-\infty <x<\infty \end{array}$$
  where *μ* and *σ* are the mean and standard deviation, respectively. Then, for any set of *N*_*i*_ values the weights *w*_*ij*_ could be computed as
  $$\begin{array}{c} w_{ij}^{G}=\frac{w_{ij}^{\prime G}}{\sum_{j=1}^{N_{i}}w_{ij}^{\prime G}},\;j=1,...,N_{i} \end{array}$$ (8)
  where
  $$\begin{array}{c} w_{ij}^{\prime G}=\frac{1}{\sigma_{N_{i}}\sqrt{2\pi }}\exp(-\frac{{\left(j-\mu_{N_{i}}\right)}^{2}}{2\sigma_{N_{i}}}) \end{array}$$ (9)
  *μ*_*N*_*i*__ and *σ*_*N*_*i*__ are the mean and standard deviation of the collection 1, 2, …, *N*_*i*_, respectively. It is easy to verify that the mean *μ*_*N*_*i*__ and the standard deviation *σ*_*N*_*i*__ could be computed as
  $$\begin{array}{c} \mu_{N_{i}}=\lambda (1+N_{i}),\;\sigma_{N_{i}}=\sqrt{\frac{1}{N_{i}}\sum_{j=1}^{N_{i}}{\left(j-\mu_{N_{i}}\right)}^{2}} \end{array}$$ (10)
  where *λ* defines the ordinal position to which we intend to assign the maximum value of weigth. If *λ* = 0.5 the weigths are symmetrical about the median ordinal position; moreover the values of $S_{ij}$ close to minimum and maximum ordinal positions had the lower values of weights. This choice of weights allowed the use of information from “most”of the values $S_{ij}$.

## Results

The X-ray diffraction analysis of *tesserae* showed the occurrence of calcite as the main mineral, while quartz was present at a low amount. The petrographic observations of thin sections confirmed that the mosaic tiles were characterized by sedimentary rocks with an isotropic sparitic/microsparitic texture and the presence of microfossils (foraminifera) and pellets (mud aggregates composed of micrite and bioclastic grains with micrite envelopes). The mineralogical component was represented by calcite crystals, which were seldom joined in twin crystals (see Fig 1, right top panel). The density, water coefficient absorption and roughness are shown in Table 1. Fig 1 (left bottom panel) shows the porosity distribution of three samples scanned with μCT (mean ± sd) over the range 0-280 μm of depth. A 3-D reconstruction of these layers is shown in Fig 1 (right bottom panel). We calculated the roughness profile from 1600 sample points for each *tessera* to determine the average roughness (*Ra*), the root mean square surface roughness (*Rq*), mean roughness depth (*Rz*) and maximum or total roughness (*Rt*). In Table 1, we report the above data with the mean density and the water absorption coefficient only for the *tesserae* for which we obtained meaningful microbial growth.

**Table 1:** Density, water absorption and roughness of *tesserae* on which the microbial strains grew.

Strain	Mean density (*g cm*^−3^)	Water absorption coefficient (*g dm*^−2^ *min*^−1/2^)	Ra	Rq	Rz	Rt
C. membranacea	2.30	1.29^-5^	1.89	2.46	10.22	14.98
C. rubescens	2.76	1.68^-5^	2.84	3.29	11.87	14.86
F. ambigua	3.74	2.12^-5^	3.26	3.87	14.57	17.99
M. diplosiphon	2.81	2.13^-5^	2.29	2.73	10.37	15.27
M. autumnalis	3.62	2.51^-5^	2.07	2.58	9.54	13.36
N. sphaerocarpa	2.42	1.40^-5^	3.15	3.89	14.27	16.72
N. commune	2.56	1.66^-5^	3.15	3.64	12.86	17.82
Plectonema sp.	2.77	2.06^-5^	2.91	3.75	16.81	24.59

All of the *tesserae* were removed from the flasks and placed in sealed chambers under controlled conditions of relative humidity, light and temperature. After two weeks, we observed that only eight microbial strains grew on the *tesserae* (Table 2). The surface covered by microbial strains presented different colors. Cyanobacteria gave the typical blue-green color observed on most *tesserae*, shifting from the brilliant green of *Nostoc commune* and *Plectonema* sp., to a more deep dark green scale observed in the *tesserae* colonized by *Microcoleus autumnalis* and *Fischerella ambigua*. However, in the case of *Calothrix membranacea*, a dominant gray color partially overlapped the blue-green observed elsewhere. To quantify the growth of the eight surviving strains (in bold in Table 2), three digital photographs of each *tessera* were captured, and the substratum coverage (mean ± sd) was calculated (Fig 2). The total covered area ranged from 19% (*Fischerella ambigua*) to 97% (*Microcoleus autumnalis*). Cyanobacteria had better growth performances, whereas the only green alga attaining a superficial coverage higher than 50% was *Coelastrella rubescens*.

**Table 2:** List of Cyanobacterial and microalgal (*) strains selected for the experiments of colonization.

Strain	Mean growth (%)	
	*t*_0_	*t*_10_
Aulosira terrestre	6.04	1.43
**Calothrix membranacea**	49.54	32.57
Chlorella vulgaris (*)	95.68	0.49
Coccomyxa solorinae-saccatae (*)	67.25	4.39
Coelastrella rubescens (*)	70.34	71.00
**Fischerella ambigua**	92.54	19.13
**Microchaete diplosiphon**	95.08	88.50
**Microcoleus autumnalis**	99.17	97.20
**Nodularia sphaerocarpa**	67.25	18.00
**Nostoc commune**	81.80	86.00
**Plectonema sp.**	85.67	88.20
Pseudococcomyxa simplex (*)	94.91	3.89
Scenedesmus communis (*)	56.37	14.92
Scytonema mirabile	13.09	9.47
Stichococcus bacillaris (*)	99.32	12.57

The substratum coverage of the microbial populations on each *tessera* was evaluated at initial time (*t*_0_) and after 10 days (*t*_10_). The eight surviving strains are highlighted in bold.

The Pearson’s correlation coefficient revealed a significant positive correlation (0.91) between the substratum coverage on mosaic *tesserae* and on the MIPs of the confocal Z-stack (Fig 2). A greater degree of correlation was observed for the reduced set of *Calothrix membranacea*, *Coelastrella rubescens*, *Fischerella ambigua*, *Nostoc commune*, and *Plectonema* sp. strains (0.97).

We compared, in a quantitative way, the spatial distribution of the strains on each slice obtained by CLS images using the dimensionless quantities in Eqs (2)–(4). In Fig 3, we observed that the substratum coverage showed a typical Gaussian shape, with reduced values at the tails corresponding to the upper and lower edges of the microbial pattern. The ratio between the characteristic linear measure of the microbial pattern and that of the slice is shown in Fig 4. Figs 3 and 4 both indicate that *N. commune* and *Plectonema* sp. had the highest values, *C. rubescens* and *M. diplosiphon* were at an intermediate level, and all of the other strains exhibited much lower values. The normalized values of the fractal dimension followed the same trend (see Fig 5). It was evident that the spatial arrangement of the phototrophic organisms on the *tesserae* could be grouped in two different types, based on Figs 3–5 and Table 2. In the first type, the architecture of the biofilm was compact, with a reduced number of empty spaces between cells or filaments, whereas in the second type, a reticulate texture allowed the presence of numerous empty volumes.

We defined these two types as compact and porous, respectively. To allow a more detailed synthesis and comparison of data obtained from the CSLM stack analyses, we aggregated the *N*_*i*_ values $A_{ij},L_{ij},D_{ij}$, for *i* = 1, …, 8, as in Eqs (5)–(7) (see S3–S5 Tables), and then we evaluated their means (see Table 3).

**Table 3:** Mean values of the aggregation operators.

Strain	$\bar{\left(A_{i}^{\max},L_{i}^{\max},D_{i}^{\max}\right)}$	$\bar{\left({\bar{A}}_{i},{\bar{L}}_{i},{\bar{D}}_{i}\right)}$	$\bar{\left(A_{i}^{w},L_{i}^{w},D_{i}^{w}\right)}$
Calothrix membranacea	0.323837	0.260432	0.275539
Coelastrella rubescens	0.398235	0.304611	0.319582
Fischerella ambigua	0.345014	0.277347	0.293566
Microchaete diplosiphon	0.423429	0.292897	0.327389
Microcoleus autumnalis	0.309799	0.276611	0.282816
Nodularia sphaerocarpa	0.347104	0.275312	0.288315
Nostoc commune	0.533988	0.389427	0.417707
Plectonema sp.	0.494467	0.36662	0.399202

Mean values of the different aggregation operators for the quantities $A_{ij},L_{ij},D_{ij}$ which refer to any microbial population (see Eqs (3)–(5)).

To better compare the structure of the strain populations by means of the quantities reported in Table 3, we rescaled the values $A_{i}^{\max},{\bar{A}}_{i},A_{i}^{w},L_{i}^{\max},{\bar{L}}_{i},L_{i}^{w},D_{i}^{\max},{\bar{D}}_{i},D_{i}^{w}$, for *i* = 1, …, 8, in such a way that they belonged to the interval [0, 1], as follows (see S6–S8 Tables)

$$\begin{array}{ccc} A_{Ni}^{\max} & = & \frac{1}{\max_{h=1,...,8}A_{h}^{\max}}A_{i}^{\max},\;{\bar{A}}_{Ni}=\frac{1}{\max_{h=1,...,8}{\bar{A}}_{h}}{\bar{A}}_{i},\;A_{Ni}^{w}=\frac{1}{\max_{h=1,...,8}A_{h}^{w}}A_{i}^{w},\\ L_{Ni}^{\max} & = & \frac{1}{\max_{h=1,...,8}L_{h}^{\max}}L_{i}^{\max},\;{\bar{L}}_{Ni}=\frac{1}{\max_{h=1,...,8}{\bar{L}}_{h}}{\bar{L}}_{i},\;L_{Ni}^{w}=\frac{1}{\max_{h=1,...,8}L_{h}^{w}}L_{i}^{w},\\ D_{Ni}^{\max} & = & \frac{1}{\max_{h=1,...,8}D_{h}^{\max}}D_{i}^{\max},\;{\bar{D}}_{Ni}=\frac{1}{\max_{h=1,...,8}{\bar{D}}_{h}}{\bar{D}}_{i},\;D_{Ni}^{w}=\frac{1}{\max_{h=1,...,8}D_{h}^{w}}D_{i}^{w}. \end{array}$$ (11)

**Fig 6:**
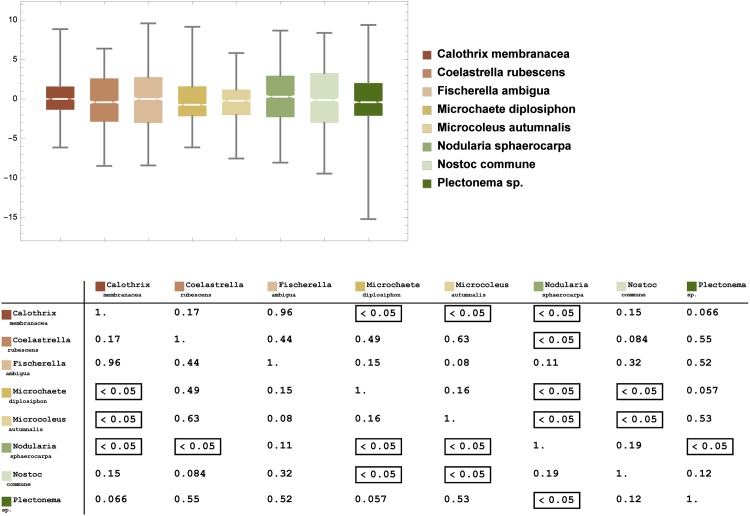
Upper panel: box and whisker plots of the roughness data. The box represents the interquartile range (IQR, lower, middle (median), and upper quartiles), whereas the whiskers above and below the box show the locations of the minimum and maximum roughness. Bottom panel: p-values of Mann-Whitney U tests at the 5% level of significance (see S1 Text). P-values above 0.05 meant that there was not enough evidence to reject the null hypothesis *H*_0_ (not framed values).

We then evaluated their means (see Table 4). Surface roughness can play a pivotal role in the colonization of lithic substrates. Characklis et al. [34] noted that the extent of microorganism colonization appeared to increase as the surface roughness increased. This was because the shear forces were diminished and the surface area was higher on rougher surfaces. The *tesserae* used in this study were from a Roman Mosaic and, due to the intrinsic characteristic of the material and very long exposure to climatic conditions, could present different values of surface roughness. A detailed quantification of this parameter for each *tessera* was necessary because the difference observed in the growth of the selected strains could depend on different surface textures and the orientation of the substrate at the microscale level. A box and whisker plot was used to summarize the roughness data (see Fig 6, upper panel). Moreover, the *Mann-Whitney U-tests* for the independent samples (each consisting of 1600 roughness values on each *tessera*) were performed (see S1 Text). For any pair of data (*data*_*i*_, *data*_*j*_), we tested the null hypothesis *H*_0_: *m*_*i*_ − *m*_*j*_ = 0 where *m*_*i*_ and *m*_*j*_ are the medians of *data*_*i*_ and *data*_*j*_, respectively, at the significance level 0.05 (see Fig 6 bottom panel).

**Table 4:** Mean values of the rescaled quantities from Table 3.

Strain	$\bar{\left(A_{N_{i}}^{\max},L_{N_{i}}^{\max},D_{N_{i}}^{\max}\right)}$	$\bar{\left({\bar{A}}_{N_{i}},{\bar{L}}_{N_{i}},{\bar{D}}_{N_{i}}\right)}$	$\bar{\left(A_{N_{i}}^{w},L_{N_{i}}^{w},D_{N_{i}}^{w}\right)}$
Calothrix membranacea	0.410930	0.423024	0.425755
Coelastrella rubescens	0.576164	0.513284	0.507068
Fischerella ambigua	0.428324	0.424578	0.430884
Microchaete diplosiphon	0.574195	0.519887	0.551082
Microcoleus autumnalis	0.374690	0.400949	0.394518
Nodularia sphaerocarpa	0.442359	0.444258	0.443263
Nostoc commune	0.988081	1.	0.996889
Plectonema sp.	0.78864	0.800178	0.82809

The data from Table 4 confirmed the same trends observed in Figs 3–5. The results of the Mann-Whitney U tests indicated that at least for five of the strains, there was no relationship between surface roughness and growth architecture (see S1 Text and Table 5). Due to the results in Table 5, we defined a *colonization index* *I*_*C*_ of each species as the mean value of quantities in Eq (11) for any fixed aggregation operator.

**Table 5:** Selected values from Table 4.

Strain	$\bar{\left(A_{N_{i}}^{\max},L_{N_{i}}^{\max},D_{N_{i}}^{\max}\right)}$	$\bar{\left({\bar{A}}_{N_{i}},{\bar{L}}_{N_{i}},{\bar{D}}_{N_{i}}\right)}$	$\bar{\left(A_{N_{i}}^{w},L_{N_{i}}^{w},D_{N_{i}}^{w}\right)}$
Calothrix membranacea	0.410930	0.423024	0.425755
Coelastrella rubescens	0.576164	0.513284	0.507068
Fischerella ambigua	0.428324	0.424578	0.430884
Nostoc commune	0.988081	1.	0.996889
Plectonema sp.	0.78864	0.800178	0.82809

## Discussion

Biofilm mats can occur at any place and on any substrate when environmental conditions permit their settlement [35], but with very different structures and features [36]. Laboratory model systems represent a useful tool to investigate the principles relevant to the microbial colonization of lithotypes. The mosaics found in archaeological sites, are generally structured as flooring and exposed to stagnation of water after abundant rainfall, followed by dry periods. Our experimental tests indicate that almost all the examined microorganism show a marked growth on *tesserae* when grown in submerged conditions. On the contrary, when the *tesserae* were exposed to the air, even under high moisture conditions, filamentous Cyanobacteria showed the best survival performances (see Table 2). Miller et al. [37] reported that various types of algae produce different engraftments on limestone substrates with low *coefficient of capillarity*, i.e. the rate at which a rock soaks up water [41]. Also the *tesserae* used in this experiment showed a low coefficient of water absorption (1.86^−5^ g dm^−2^ min^−1/2^). Since Roman mosaics were often placed on the border of soils, rain water continuously drains soil particles and nutrients onto the *tesserae*. Then, our test conditions, in a closed environmental system, simulate a model of bare microenvironmental colonization under replete nutrient conditions. All these factors, combined with the intrinsic characteristics of the substrate, has favored the growth of filamentous Cyanobacteria [38, 39].

Following the suggestion of Murdoch and Dodds [40] in that microscale features of the substrate may affect the microbial growth also at the macroscale, we compared the results obtained with the image analysis of the whole *tesserae* with the MIP images. Our data supported the finding that the microscale assemblage of a monospecific biofilm was mirrored by its macroscale distribution on the substratum (Pearson’s correlation coefficient over 0.9). The biofilm image analysis of individual components (area, perimeter, and fractal dimension) of the Z-stack CLS was found to be a good tool to assess the ability of the colonization of a stone surface by a monospecific microbial population, and it furnished a clear picture of the early stages of settlement on a substrate. Cyanobacteria had a better ability to colonize lithic substrates, whereas the only Chlorophyta that had good growth on the *tesserae* was *Coelastrella rubescens*. The analysis of a single slice of a Z-stack showed that all of the strains tended to occupy larger areas in the middle layers of the biofilm, exhibiting reduced growth in both the upper and bottom layers of the structure. In our experiments, the cells were exposed to 150 *μ*mol photon m^−2^ s^−1^ irradiance from above, and light was probably limiting in the bottom layer of both the porous and compact biofilms. It is conceivable that the biofilm bottom layer of phototrophic organisms could represent a region of metabolic resting stage, as observed in bacterial biofilms, resulting in the physiological stratification of the mat layers. In addition, the upper region of the biofilm could show physiological specialization. Serra et al. [42] suggested that the temporal physiological changes observed in bacterial liquid culture correspond to spatial order changes in biofilm communities. It is well known that, under a circadian rhythm, phototrophic organisms carry out photosynthesis during the day, concentrating on DNA duplication and cell division at night. In the case of a biofilm under a continuous dim light, the upper layers, where light is not limited, are involved in photosynthesis. The middle layers, which contain actively dividing cells, support the biomass production of the whole community. Conversely, the comparison of the patterns lining the microorganism diffusion on each slice of a Z-stack shed light on at least two growth styles (compact and dense or porous) we observed. For each biofilm, the characteristic of compact or porous was highlighted by using the data on the areas, perimeters and fractal dimensions of each single slice of the Z-stacks by means of Eqs (2)–(4). In particular, Eq (3) is a novel quantity specifically developed for this study, whereas the others are standard indices of biofilm growth and complexity. The quantities in Eqs (2)–(4) indicated that the microorganisms showing values close to zero had a strong characteristic of a porous colonizer. Those with values close to 1 had a strong characteristic of a compact and dense colonizer. The aggregation and rescaling of these quantities confirmed the same characterization independently of the statistical aggregations (Eqs (5)–(7)). Therefore, this characterization is robust from a mathematical and statistical point of view. It is well known that roughness has a major role in determining the colonization of a substrate [40, 43]. The statistical analysis confirmed that, in our experimental conditions that were common to all of the strains, at least in five out of the eight selected species, the growth on the *tesserae* was not related to roughness and should be considered an intrinsic character of each strain. The mode of growth, compact or porous, inevitably dictated the 3D structures of the biofilm during the early stages of colonization. In porous growth (e.g., *Fischerella ambigua*), the presence of a reticulate structure allowed air and water entrapment in the mat layers, whereas a compact structure (e.g., *Nostoc commune*) may have affected the local concentration of oxygen and also had an influence on the distribution of humidity within the layers [44]. Moreover, a porous biofilm could host different biological organisms (algae, fungi, bacteria, etc.) into its shortcomings/gaps, creating the conditions for the establishment of a strong heterogeneity. On the contrary, a compact biofilm resulted in a more homogeneous mat, where potential hosts were mainly confined to the superficial layers. According to Table 5, the colonization index of a porous microbial population was lower than 0.5, for compact microbial structure ranges in 0.7-1, whereas values between 0.5 and 0.7 referred to species with intermediate behavior. In our case, *Calothrix membranacea* and *Fischerella ambigua* could be considered porous colonizers, *Nostoc commune* and *Plectonema* sp. were compact colonizers, and *Coelastrella rubescens* occupied an intermediate position.

## Conclusion

This paper represents a first step toward understanding and characterizing the colonization of phototrophic microorganism on stone surfaces, particularly on mosaic *tesserae*. Cyanobacteria and microalgae seemed to share the same modality of colonization described for bacterial populations, dense or porous, and showed different degrees of substratum coverage and architecture that were peculiar to each strain. The statistical analysis confirmed that the microbial population grew in homogeneous conditions (light, temperature, RH, petrographic features, and surface roughness of the substrate). Therefore, the different colonization processes could be considered an intrinsic characteristic of each strain. The proposed index *I*_*C*_ represented a sensible tool to describe, in a quantitative way, the pioneering attitude of photosynthetic microorganisms under laboratory conditions. This index could be extended to characterize multispecies biofilms composed of autotrophic and heterotrophic organisms. In future studies, we want to verify how the modality of colonization is influenced by the presence of other organisms and/or changing environmental conditions.

## Supporting Information

S1 Text Mann-Whitney U tests for median differences of perimeter, area, and fractal dimension data. The values of area, perimeter, and fractal dimension relative to colonized areas obtained from the stacked CLS images (for each microbial population, we had three sets of *N*_*i*_ data, where *N*_*i*_ is the number of CLS images for *i*–th algal population (*i* = 1, …, 8) (see S1 Table, first column) were summarized in the box and whisker plots (see S1–S3 Figs, upper panels). Moreover, these three sets of data were compared pairwise to test their statistical indistinguishability using the Mann-Whitney U-tests at significance level 0.05 (see S1–S3 Figs, bottom panels). The statistical analysis was performed using the routine *LocationTest* of the software *Mathematica*^®^ (ver. 10.4 Wolfram).
(PDF)

S1 Fig Perimeter data: Box and Whisker summary plots; p-values of Mann-Whitney U test. (upper panel) Box and Whisker summary plots for the three sets of the perimeter values. Each box represents the interquartile range (IQR, lower, middle (median), and upper quartiles), whereas whiskers above and below each box show the locations of the minimum and maximum roughness; (bottom panel) p-values of Mann-Whitney U test at the 5% level of significance. P-values above 0.05 means that there is not enough evidence to reject the null hypotheses *H*_0_: *m*_*i*_ − *m*_*j*_ = 0 where *m*_*i*_ and *m*_*j*_ are the medians of *P*_*i*_ and *P*_*j*_ data.
(PDF)

S2 Fig Area data: Box and Whisker summary plots; p-values of Mann-Whitney U test. (upper panel) Box and Whisker summary plots for the three sets of the area values. Each box represents the interquartile range (IQR, lower, middle (median), and upper quartiles, whereas whiskers above and below each box show the locations of the minimum and maximum roughness; (bottom panel) p-values of Mann-Whitney U test at the 5% level of significance (see S1 Text). P-values above 0.05 means that there is not enough evidence to reject the null hypotheses *H*_0_: *m*_*i*_ − *m*_*j*_ = 0 where *m*_*i*_ and *m*_*j*_ are the medians of *A*_*i*_ and *A*_*j*_ data.
(PDF)

S3 Fig Fractal dimension: Box and Whisker summary plots; p-values of Mann-Whitney U test. (upper panel) Box and Whisker summary plots for the three sets of the fractal dimension values. Each box represents the interquartile range (IQR, lower, middle (median), and upper quartiles, whereas whiskers above and below each box show the locations of the minimum and maximum fractal dimension; (bottom panel) p-values of Mann-Whitney U test at the 5% level of significance (see S1 Text). P-values above 0.05 means that there is not enough evidence to reject the null hypotheses *H*_0_: *m*_*i*_ − *m*_*j*_ = 0 where *m*_*i*_ and *m*_*j*_ are the medians of *D*_*Bi*_ and *D*_*Bj*_ data.
(PDF)

S4 Fig Area and perimeter. Area and perimeter (mean±SD of three sampling points) relative to colonized areas obtained from confocal Z-stack images.
(PDF)

S5 Fig Fractal dimension. Fractal dimension (mean±SD of three sampling points) relative to colonized areas obtained from confocal Z-stack images.
(PDF)

S1 Table Z-stacks. Summary of main characteristics of the Z-stacks.
(PDF)

S2 Table Fractal dimensions. Fractal dimensions *D*_*B*_ and the coefficients of determination *R*^2^ relative to colonized areas obtained from each slice of confocal Z-stacks at the three sample points.
(PDF)

S3 TableMaximum operator.Maximum operator of aggregation for the quantities $A_{ij},L_{ij},D_{ij}$.(PDF)

S4 TableAverage operator.Average operator of aggregation for the quantities $A_{ij},L_{ij},D_{ij}$.(PDF)

S5 TableWeighted average operator.Weighted average operator of aggregation for the quantities $A_{ij},L_{ij},D_{ij}$.(PDF)

S6 TableRescaled values of quantities $A_{i}^{\max},L_{i}^{\max},D_{i}^{\max}$.(PDF)

S7 TableRescaled values of quantities ${\bar{A}}_{Ni},{\bar{L}}_{Ni},{\bar{D}}_{Ni}$.(PDF)

S8 TableRescaled values of quantities $A_{i}^{w},L_{i}^{w},D_{i}^{w}$.(PDF)
