# Supplementary material for: Weathering of a Roman Mosaic—A Biological and Quantitative Study on In Vitro Colonization of Calcareous Tesserae by Phototrophic Microorganisms
Source: PLoS One. 2016 Oct 26;11(10):e0164487. doi: 10.1371/journal.pone.0164487 (PMC5082677; doi:10.1371/journal.pone.0164487)
Supplement: S1 Text — The values of area, perimeter, and fractal dimension relative to colonized areas obtained from the stacked CLS images (for each microbial population, we had three sets of Ni data, where Ni is the number of CLS images for i–th algal population (i = 1, …, 8) (see S1 Table, first column) were summarized in the box and whisker plots (see S1–S3 Figs, upper panels). Moreover, these three sets of data were compared pairwise to test their statistical indistinguishability using the Mann-Whitney U-tests at significance level 0.05 (see S1–S3 Figs, bottom panels). The statistical analysis was performed using the routine LocationTest of the software Mathematica® (ver. 10.4 Wolfram). (PDF) [file pone.0164487.s001.pdf]

S1 Text

Mann-Whitney U tests for median differences of perimeter, area, and fractal dimension data. The values of area, perimeter, and fractal dimension relative to colonized areas obtained from the stacked CLSM images (for each microbial population, we had three sets of N_i data, where N_i is the number of CLSM images for $i-$th algal population ($i = 1, ..., 8$) (see S1 Table, first column) were summarized in the box and whisker plots (see S1 Fig–S3 Fig, upper panels). Moreover, these three sets of data were compared pairwise to test their statistical indistinguishability using the Mann-Whitney U-tests at significance level 0.05 (see S1 Fig–S3 Fig, bottom panels). The statistical analysis was performed using the routine *LocationTest* of the software *Mathematica*® (ver. 10.4 Wolfram).
