# Supplementary material for: Weathering of a Roman Mosaic—A Biological and Quantitative Study on In Vitro Colonization of Calcareous Tesserae by Phototrophic Microorganisms
Source: PLoS One. 2016 Oct 26;11(10):e0164487. doi: 10.1371/journal.pone.0164487 (PMC5082677; doi:10.1371/journal.pone.0164487)
Supplement: S1 Fig — (upper panel) Box and Whisker summary plots for the three sets of the perimeter values. Each box represents the interquartile range (IQR, lower, middle (median), and upper quartiles), whereas whiskers above and below each box show the locations of the minimum and maximum roughness; (bottom panel) p-values of Mann-Whitney U test at the 5% level of significance. P-values above 0.05 means that there is not enough evidence to reject the null hypotheses H0: mi − mj = 0 where mi and mj are the medians of Pi and Pj data. (PDF) [file pone.0164487.s002.pdf]

S1 Fig

Perimeter data: Box and Whisker summary plots; p-values of Mann-Whitney U test. (upper panel) Box and Whisker summary plots for the three sets of the perimeter values. Each box represents the interquartile range (IQR, lower, middle (median), and upper quartiles), whereas whiskers above and below each box show the locations of the minimum and maximum roughness; (bottom panel) p-values of Mann-Whitney U test at the 5% level of significance. P-values above 0.05 means that there is not enough evidence to reject the null hypotheses $H_0 : m_i - m_j = 0$ where m_i and m_j are the medians of P_i and P_j data.

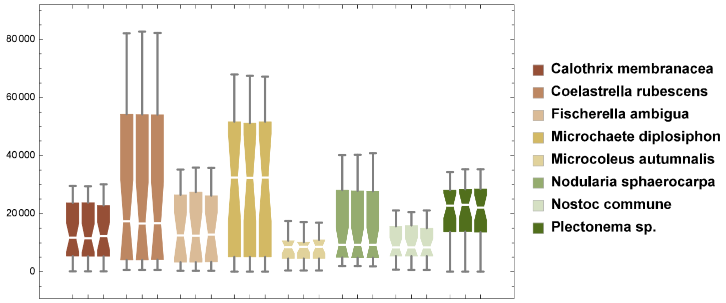

	p - value (P_1, P_2)	p - value (P_1, P_3)	p - value (P_2, P_3)
Calothrix membranacea	0.946	0.966	0.927
Coelastrella rubescens	1.	0.966	0.944
Fischerella ambigua	0.972	0.953	0.935
Microchaete diplosiphon	0.959	0.992	0.992
Microcoleus autumnalis	0.884	0.93	0.748
Nodularia sphaerocarpa	0.988	0.988	0.996
Nostoc commune	0.934	0.891	0.964
Plectonema sp.	0.851	0.954	0.98

S1 Fig
