# Supplementary material for: Weathering of a Roman Mosaic—A Biological and Quantitative Study on In Vitro Colonization of Calcareous Tesserae by Phototrophic Microorganisms
Source: PLoS One. 2016 Oct 26;11(10):e0164487. doi: 10.1371/journal.pone.0164487 (PMC5082677; doi:10.1371/journal.pone.0164487)
Supplement: S2 Fig — (upper panel) Box and Whisker summary plots for the three sets of the area values. Each box represents the interquartile range (IQR, lower, middle (median), and upper quartiles, whereas whiskers above and below each box show the locations of the minimum and maximum roughness; (bottom panel) p-values of Mann-Whitney U test at the 5% level of significance (see S1 Text). P-values above 0.05 means that there is not enough evidence to reject the null hypotheses H0: mi − mj = 0 where mi and mj are the medians of Ai and Aj data. (PDF) [file pone.0164487.s003.pdf]

## S2 Fig

**Area data: Box and Whisker summary plots; p-values of Mann-Whitney U test.** (upper panel) Box and Whisker summary plots for the three sets of the area values. Each box represents the interquartile range (IQR, lower, middle (median), and upper quartiles, whereas whiskers above and below each box show the locations of the minimum and maximum roughness; (bottom panel) p-values of Mann-Whitney U test at the 5% level of significance (see S1 Text). P-values above 0.05 means that there is not enough evidence to reject the null hypotheses $H_0 : m_i - m_j = 0$ where $m_i$ and $m_j$ are the medians of $A_i$ and $A_j$ data.

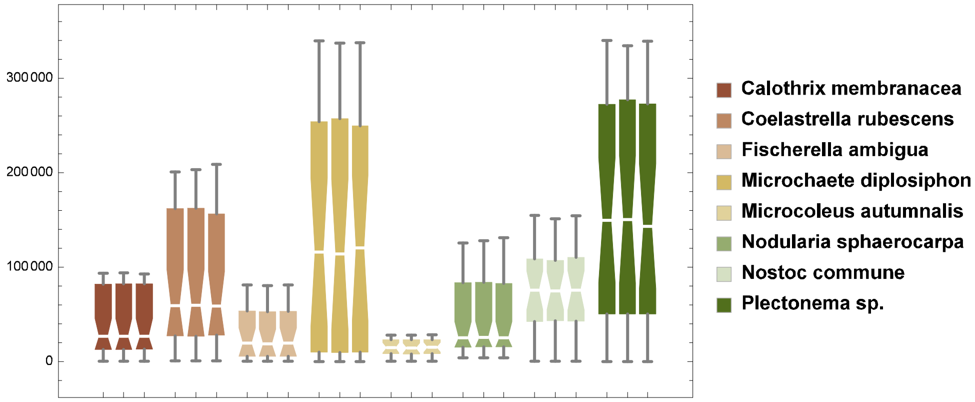


| | p - value ($A_1$, $A_2$) | p - value ($A_1$, $A_3$) | p - value ($A_2$, $A_3$) |
|---|---|---|---|
| Calothrix membranacea | 0.995 | 0.995 | 0.976 |
| Coelastrella rubescens | 0.944 | 0.978 | 1. |
| Fischerella ambigua | 0.953 | 0.991 | 0.916 |
| Microchaete diplosiphon | 0.942 | 0.91 | 0.893 |
| Microcoleus autumnalis | 0.953 | 0.907 | 0.861 |
| Nodularia sphaerocarpa | 0.938 | 0.938 | 0.938 |
| Nostoc commune | 0.876 | 0.964 | 0.92 |
| Plectonema sp. | 0.993 | 0.967 | 0.967 |

S2 Fig
