# Supplementary material for: Weathering of a Roman Mosaic—A Biological and Quantitative Study on In Vitro Colonization of Calcareous Tesserae by Phototrophic Microorganisms
Source: PLoS One. 2016 Oct 26;11(10):e0164487. doi: 10.1371/journal.pone.0164487 (PMC5082677; doi:10.1371/journal.pone.0164487)
Supplement: S3 Fig — (upper panel) Box and Whisker summary plots for the three sets of the fractal dimension values. Each box represents the interquartile range (IQR, lower, middle (median), and upper quartiles, whereas whiskers above and below each box show the locations of the minimum and maximum fractal dimension; (bottom panel) p-values of Mann-Whitney U test at the 5% level of significance (see S1 Text). P-values above 0.05 means that there is not enough evidence to reject the null hypotheses H0: mi − mj = 0 where mi and mj are the medians of DBi and DBj data. (PDF) [file pone.0164487.s004.pdf]

S3 Fig

Fractal dimension: Box and Whisker summary plots; p-values of Mann-Whitney U test. (upper panel) Box and Whisker summary plots for the three sets of the fractal dimension values. Each box represents the interquartile range (IQR, lower, middle (median), and upper quartiles, whereas whiskers above and below each box show the locations of the minimum and maximum fractal dimension; (bottom panel) p-values of Mann-Whitney U test at the 5% level of significance (see S1 Text). P-values above 0.05 means that there is not enough evidence to reject the null hypotheses $H_0 : m_i - m_j = 0$ where m_i and m_j are the medians of D_{Bi} and D_{Bj} data.

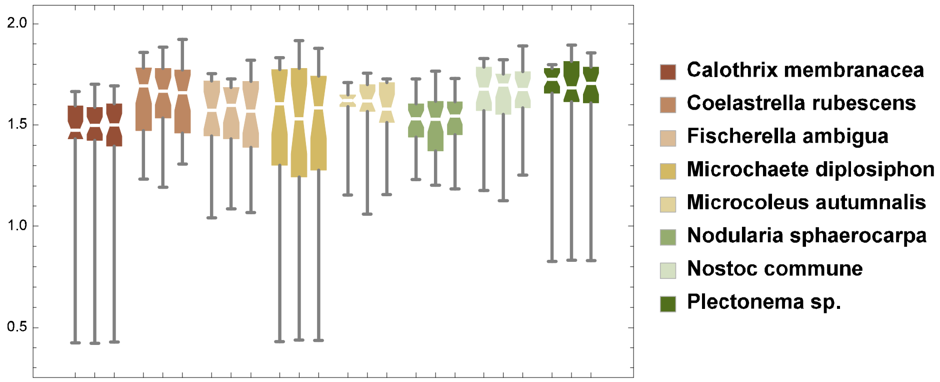

	p - value (D_{B1}, D_{B2})	p - value (D_{B1}, D_{B3})	p - value (D_{B2}, D_{B3})
Calothrix membranacea	0.985	0.773	0.83
Coelastrella rubescens	0.767	0.855	0.989
Fischerella ambigua	0.681	0.879	0.63
Microchaete diplosiphon	0.959	0.797	0.734
Microcoleus autumnalis	0.704	0.793	0.448
Nodularia sphaerocarpa	0.856	0.736	0.636
Nostoc commune	0.735	0.934	0.848
Plectonema sp.	0.688	0.7	0.572

S3 Fig
