# Supplementary material for: Weathering of a Roman Mosaic—A Biological and Quantitative Study on In Vitro Colonization of Calcareous Tesserae by Phototrophic Microorganisms
Source: PLoS One. 2016 Oct 26;11(10):e0164487. doi: 10.1371/journal.pone.0164487 (PMC5082677; doi:10.1371/journal.pone.0164487)
Supplement: S4 Fig — Area and perimeter (mean±SD of three sampling points) relative to colonized areas obtained from confocal Z-stack images. (PDF) [file pone.0164487.s005.pdf]

S4 Fig

Area and perimeter. Area and perimeter (mean±SD of three sampling points) relative to colonized areas obtained from confocal Z-stack images.

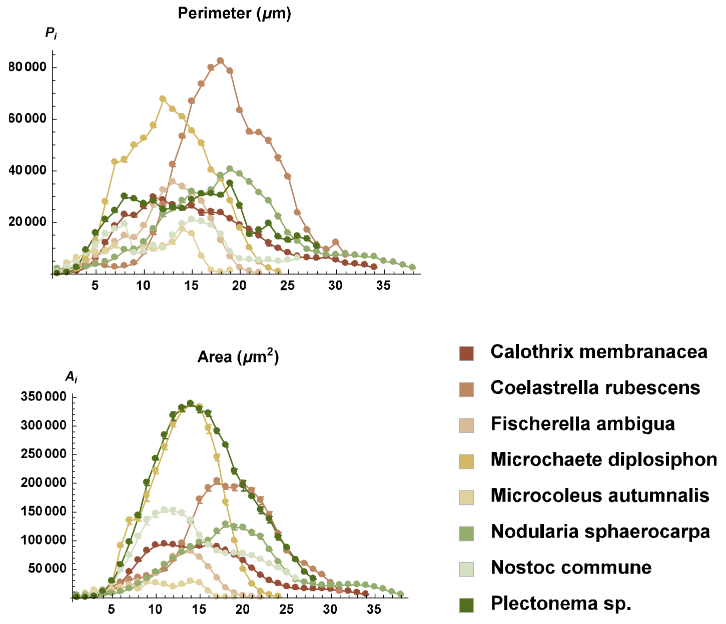

S4 Fig
