# Supplementary material for: Weathering of a Roman Mosaic—A Biological and Quantitative Study on In Vitro Colonization of Calcareous Tesserae by Phototrophic Microorganisms
Source: PLoS One. 2016 Oct 26;11(10):e0164487. doi: 10.1371/journal.pone.0164487 (PMC5082677; doi:10.1371/journal.pone.0164487)
Supplement: S5 Fig — Fractal dimension (mean±SD of three sampling points) relative to colonized areas obtained from confocal Z-stack images. (PDF) [file pone.0164487.s006.pdf]

S5 Fig

Fractal dimension. Fractal dimension (mean±SD of three sampling points) relative to colonized areas obtained from confocal Z-stack images.

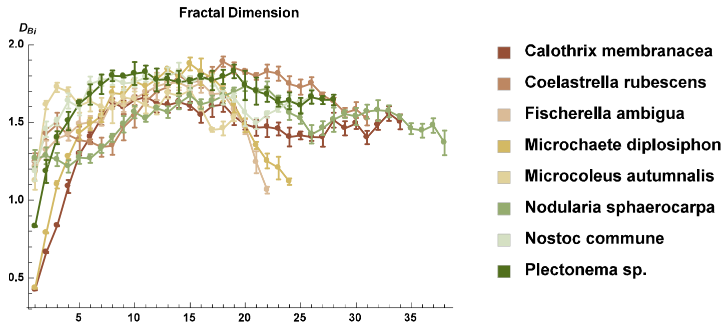

S5 Fig
