# Supplementary material for: Weathering of a Roman Mosaic—A Biological and Quantitative Study on In Vitro Colonization of Calcareous Tesserae by Phototrophic Microorganisms
Source: PLoS One. 2016 Oct 26;11(10):e0164487. doi: 10.1371/journal.pone.0164487 (PMC5082677; doi:10.1371/journal.pone.0164487)
Supplement: S1 Table — Summary of main characteristics of the Z-stacks. (PDF) [file pone.0164487.s007.pdf]

S1 Table

Z-stacks. Summary of main characteristics of the Z-stacks.

	Slice number	Slice area (μm^2)
Calothrix membranacea	34	770480.17
Coelastrella rubescens	31	1007052.39
Fischerella ambigua	22	558786.15
Microchaete diplosiphon	24	1090938.47
Microcoleus autumnalis	19	562500.
Nodularia sphaerocarpa	38	808129.08
Nostoc commune	26	272734.62
Plectonema sp.	28	671088.64

S1 Table
