# Supplementary material for: Weathering of a Roman Mosaic—A Biological and Quantitative Study on In Vitro Colonization of Calcareous Tesserae by Phototrophic Microorganisms
Source: PLoS One. 2016 Oct 26;11(10):e0164487. doi: 10.1371/journal.pone.0164487 (PMC5082677; doi:10.1371/journal.pone.0164487)
Supplement: S2 Table — Fractal dimensions DB and the coefficients of determination R2 relative to colonized areas obtained from each slice of confocal Z-stacks at the three sample points. (PDF) [file pone.0164487.s008.pdf]

S2 Table

Fractal dimensions. Fractal dimensions D_B and the coefficients of determination R^2 relative to colonized areas obtained from each slice of confocal Z-stacks at the three sample points.

D_B	R^2	D_B	R^2	D_B	R^2	D_B	R^2	D_B	R^2	D_B	R^2	D_B	R^2	D_B	R^2
0.423	0.912	1.234	0.99	1.211	0.998	0.429	0.936	1.156	0.988	1.271	0.987	1.177	0.998	0.827	0.98
0.669	0.98	1.386	0.993	1.304	0.999	0.79	0.985	1.642	0.996	1.309	0.991	1.4	0.999	1.176	0.995
0.832	0.988	1.492	0.992	1.42	0.998	1.073	0.994	1.697	0.999	1.286	0.992	1.477	1.	1.36	0.998
1.091	0.991	1.403	0.986	1.456	1.	1.296	0.997	1.664	0.998	1.262	0.995	1.583	0.999	1.511	0.999
1.295	0.995	1.394	0.988	1.491	1.	1.412	0.999	1.592	0.998	1.231	0.996	1.648	1.	1.617	1.
1.442	0.998	1.387	0.994	1.526	0.999	1.498	0.999	1.624	0.998	1.294	0.999	1.74	0.999	1.705	0.999
1.531	0.999	1.324	0.995	1.521	0.999	1.58	0.999	1.62	0.997	1.319	0.999	1.818	0.999	1.755	0.999
1.601	0.999	1.382	0.995	1.578	0.998	1.646	0.999	1.59	0.998	1.419	0.999	1.828	0.999	1.783	0.999
1.635	0.999	1.466	0.996	1.62	0.997	1.704	0.999	1.646	0.998	1.472	1.	1.798	0.999	1.798	0.999
1.664	0.999	1.555	0.997	1.674	0.997	1.743	0.999	1.638	0.997	1.539	1.	1.777	0.999	1.796	0.999
1.654	0.999	1.614	0.998	1.717	0.998	1.776	0.999	1.609	0.998	1.549	0.999	1.774	0.998	1.775	0.999
1.632	0.998	1.693	0.999	1.74	0.998	1.808	0.999	1.621	0.998	1.588	0.999	1.787	0.998	1.767	0.999
1.614	0.997	1.743	0.999	1.754	0.999	1.831	0.999	1.648	0.999	1.594	1.	1.792	0.999	1.766	0.999
1.594	0.998	1.773	1.	1.752	0.999	1.832	0.999	1.695	0.999	1.605	1.	1.803	0.999	1.771	0.998
1.597	0.999	1.794	0.999	1.748	0.999	1.833	0.999	1.709	0.999	1.629	0.999	1.801	0.999	1.781	0.998
1.596	0.999	1.822	0.999	1.731	0.998	1.823	1.	1.602	0.998	1.646	0.999	1.776	0.998	1.787	0.998
1.59	0.999	1.847	0.999	1.67	0.998	1.772	0.999	1.445	0.993	1.679	0.999	1.742	0.998	1.799	0.999
1.578	0.999	1.858	0.999	1.639	0.997	1.705	1.	1.453	0.994	1.71	1.	1.706	0.998	1.798	0.999
1.552	0.999	1.859	0.999	1.565	0.995	1.63	1.	1.547	0.992	1.727	1.	1.645	0.998	1.779	0.999
1.525	0.999	1.839	1.	1.446	0.993	1.519	0.999			1.724	1.	1.565	0.998	1.74	0.999
1.523	0.999	1.811	1.	1.256	0.991	1.374	0.998			1.698	1.	1.507	0.999	1.709	0.999
1.478	0.998	1.784	1.	1.041	0.99	1.305	0.996			1.666	1.	1.534	0.998	1.657	1.
1.446	0.998	1.781	1.			1.191	0.993			1.647	1.	1.569	0.999	1.659	1.
1.409	0.998	1.779	1.			1.105	0.984			1.588	0.999	1.594	0.999	1.656	1.
1.432	0.995	1.776	1.							1.493	0.999	1.616	0.998	1.666	1.
1.39	0.995	1.75	1.							1.442	0.998	1.626	0.999	1.666	1.
1.383	0.997	1.696	1.							1.454	0.998			1.65	0.999
1.459	0.997	1.628	1.							1.528	0.999			1.678	0.999
1.457	0.997	1.572	1.							1.531	0.998				
1.454	0.998	1.636	0.999							1.521	0.997				
1.437	0.998	1.55	0.997							1.536	0.997				
1.47	0.998									1.555	0.997				
1.509	0.996									1.561	0.997				
1.463	0.995									1.527	0.997				
										1.501	0.996				
										1.481	0.996				
										1.439	0.996				
										1.363	0.991				

S2 Table (panel a)

D_B	R^2	D_B	R^2	D_B	R^2	D_B	R^2	D_B	R^2	D_B	R^2	D_B	R^2	D_B	R^2
0.421	0.91	1.19	0.909	1.2	1.	0.436	0.893	1.06	0.979	1.21	0.911	1.13	0.983	0.833	0.898
0.66	0.972	1.46	0.903	1.27	0.998	0.785	0.94	1.59	0.984	1.22	0.908	1.48	0.988	1.26	0.914
0.838	0.979	1.56	0.906	1.38	0.983	1.11	0.942	1.76	0.988	1.21	0.915	1.52	0.932	1.44	0.916
1.13	0.984	1.39	0.9	1.43	0.947	1.28	0.941	1.72	0.989	1.2	0.922	1.65	0.991	1.57	0.919
1.29	0.985	1.3	0.897	1.56	0.994	1.41	0.954	1.66	0.99	1.31	0.91	1.55	0.953	1.65	0.895
1.38	0.985	1.36	0.914	1.52	0.997	1.45	0.945	1.58	0.988	1.27	0.92	1.7	0.995	1.73	0.913
1.48	0.986	1.29	0.914	1.5	0.985	1.5	0.954	1.65	0.993	1.36	0.914	1.75	0.983	1.68	0.914
1.66	0.988	1.28	0.914	1.67	0.987	1.73	0.945	1.68	0.995	1.37	0.926	1.75	0.996	1.78	0.904
1.55	0.985	1.56	0.907	1.65	0.991	1.71	0.942	1.62	0.996	1.45	0.937	1.75	0.99	1.8	0.904
1.66	0.999	1.6	0.913	1.59	0.996	1.79	0.946	1.71	0.984	1.61	0.831	1.72	0.984	1.89	0.916
1.7	0.981	1.66	0.908	1.73	0.991	1.75	0.944	1.67	0.988	1.51	0.917	1.82	0.989	1.84	0.909
1.56	0.993	1.76	0.921	1.64	0.989	1.76	0.953	1.56	0.988	1.58	0.919	1.79	1.	1.85	0.909
1.66	0.992	1.7	0.914	1.7	0.984	1.84	0.942	1.71	0.999	1.52	1.	1.75	0.996	1.82	0.904
1.63	0.984	1.79	0.837	1.71	0.991	1.8	0.95	1.61	0.998	1.6	0.991	1.77	1.	1.68	0.907
1.59	1.	1.72	0.913	1.72	0.996	1.92	0.949	1.73	0.994	1.73	0.912	1.71	0.987	1.77	0.908
1.55	0.995	1.76	0.904	1.71	0.998	1.91	0.863	1.59	1.	1.58	0.911	1.8	0.983	1.79	0.913
1.54	1.	1.76	0.906	1.66	0.987	1.77	0.946	1.44	0.994	1.62	0.927	1.81	0.99	1.81	0.919
1.6	0.996	1.89	0.92	1.68	0.996	1.8	1.	1.51	0.98	1.69	0.906	1.65	0.987	1.84	0.91
1.59	0.997	1.88	0.904	1.6	0.987	1.56	0.904	1.53	0.989	1.67	0.833	1.69	0.999	1.85	0.919
1.46	0.997	1.83	0.894	1.44	0.99	1.49	0.957			1.77	0.996	1.48	0.996	1.83	0.906
1.42	0.996	1.81	0.983	1.17	0.982	1.35	0.955			1.7	0.876	1.45	0.994	1.61	0.913
1.53	0.994	1.83	0.842	1.09	0.976	1.21	0.944			1.76	0.931	1.55	0.992	1.65	0.988
1.42	0.986	1.79	0.973			1.14	0.943			1.67	0.979	1.6	0.994	1.6	0.968
1.46	0.987	1.69	0.839			1.14	0.938			1.51	0.92	1.68	0.985	1.68	1.
1.43	0.984	1.73	0.91							1.56	0.922	1.66	0.991	1.59	0.86
1.44	0.984	1.79	0.985							1.36	0.911	1.71	0.989	1.62	0.996
1.36	0.994	1.67	0.946							1.42	0.91			1.67	0.903
1.53	0.979	1.62	0.998							1.44	0.911			1.65	0.918
1.51	0.983	1.53	1.							1.58	0.921				
1.5	0.998	1.57	0.918							1.49	0.914				
1.36	0.991	1.55	0.91							1.55	0.923				
1.46	0.989									1.65	0.916				
1.6	0.991									1.62	0.921				
1.49	0.984									1.55	0.918				
										1.43	0.916				
										1.4	0.917				
										1.53	0.91				
										1.3	0.92				

S2 Table (panel b)

D_B	R^2	D_B	R^2	D_B	R^2	D_B	R^2	D_B	R^2	D_B	R^2	D_B	R^2	D_B	R^2
0.428	0.902	1.31	0.913	1.23	0.995	0.435	0.869	1.16	0.992	1.21	0.906	1.25	0.971	0.831	0.911
0.664	0.97	1.43	0.908	1.39	0.989	0.792	0.908	1.61	0.997	1.22	0.903	1.49	0.978	1.12	0.932
0.835	0.98	1.41	0.913	1.38	0.995	1.12	0.918	1.73	0.998	1.21	0.9	1.52	0.998	1.4	0.932
1.05	0.99	1.46	0.898	1.38	0.902	1.27	0.909	1.71	0.991	1.2	0.903	1.68	0.981	1.45	0.938
1.31	0.99	1.47	0.898	1.42	0.924	1.49	0.918	1.57	0.983	1.31	0.914	1.58	1.	1.61	1.
1.39	0.999	1.38	0.919	1.46	0.987	1.52	0.914	1.71	0.99	1.27	0.915	1.8	0.973	1.61	0.939
1.57	0.999	1.41	0.909	1.48	1.	1.54	0.927	1.53	0.998	1.36	0.902	1.75	0.981	1.8	0.945
1.63	0.995	1.39	0.917	1.67	0.993	1.69	0.923	1.49	1.	1.37	0.917	1.76	0.97	1.84	0.94
1.6	0.998	1.39	0.923	1.56	0.982	1.63	0.922	1.72	0.997	1.45	0.844	1.71	0.976	1.79	0.945
1.61	0.982	1.46	0.915	1.71	0.994	1.76	0.913	1.6	0.999	1.61	0.967	1.77	0.965	1.75	0.932
1.64	0.985	1.64	0.918	1.63	0.992	1.68	0.925	1.56	0.994	1.51	0.901	1.77	0.977	1.85	0.928
1.69	0.986	1.65	0.922	1.82	0.988	1.77	0.915	1.57	0.997	1.58	0.913	1.71	0.981	1.7	0.946
1.56	0.987	1.74	0.919	1.81	0.994	1.85	0.919	1.71	0.99	1.52	0.994	1.86	0.968	1.75	0.928
1.67	0.997	1.85	0.864	1.8	0.999	1.75	0.927	1.71	1.	1.6	0.952	1.89	0.974	1.83	0.932
1.64	0.993	1.76	0.913	1.7	0.996	1.88	0.925	1.68	0.999	1.73	0.913	1.75	0.98	1.75	0.931
1.5	0.982	1.73	0.917	1.82	0.99	1.74	0.834	1.58	0.997	1.58	0.917	1.78	0.976	1.81	0.934
1.68	0.987	1.83	0.923	1.71	0.994	1.82	0.92	1.47	0.981	1.62	0.919	1.76	0.967	1.7	0.933
1.67	0.983	1.92	0.913	1.74	0.997	1.64	1.	1.42	0.985	1.69	0.955	1.62	0.98	1.75	0.934
1.48	0.998	1.81	0.914	1.57	0.989	1.65	0.854	1.5	0.996	1.67	0.981	1.65	0.97	1.86	0.937
1.48	1.	1.81	0.893	1.53	0.981	1.43	0.915			1.77	1.	1.65	0.972	1.64	0.93
1.45	0.99	1.77	0.934	1.31	0.988	1.33	0.923			1.7	0.848	1.52	0.984	1.78	0.929
1.4	0.994	1.87	0.925	1.07	0.988	1.24	0.924			1.76	0.872	1.57	0.982	1.73	0.863
1.5	0.987	1.86	0.932			1.29	0.905			1.67	0.907	1.58	0.984	1.63	0.99
1.35	0.991	1.77	0.976			1.11	0.912			1.51	0.906	1.62	0.966	1.58	0.9
1.39	0.996	1.68	0.94							1.56	0.919	1.62	0.974	1.57	0.914
1.39	0.983	1.71	0.964							1.36	0.903	1.67	0.98	1.7	0.909
1.47	0.997	1.66	0.958							1.42	0.903			1.62	0.935
1.55	0.998	1.66	0.829							1.44	0.912			1.61	0.937
1.42	0.988	1.59	1.							1.58	0.911				
1.52	0.992	1.54	0.92							1.49	0.909				
1.42	0.983	1.47	0.916							1.55	0.902				
1.52	0.983									1.65	0.9				
1.56	0.993									1.62	0.908				
1.56	0.982									1.55	0.904				
										1.43	0.903				
										1.4	0.914				
										1.53	0.907				
										1.3	0.906				

Calothrix membranacea; Coelastrella rubescens; Fischerella ambigua; Microchaete diplosiphon; Microcoleus autumnalis; Nodularia sphaerocarpa; Nostoc commune; Plectonema sp.

S2 Table (panel c)
