# Supplementary material for: Weathering of a Roman Mosaic—A Biological and Quantitative Study on In Vitro Colonization of Calcareous Tesserae by Phototrophic Microorganisms
Source: PLoS One. 2016 Oct 26;11(10):e0164487. doi: 10.1371/journal.pone.0164487 (PMC5082677; doi:10.1371/journal.pone.0164487)
Supplement: S3 Table — Maximum operator of aggregation for the quantities Aij,Lij,Dij. (PDF) [file pone.0164487.s009.pdf]

S3 Table

Maximum operator. Maximum operator of aggregation for the quantities $\mathcal{A}_{ij}, \mathcal{L}_{ij}, \mathcal{D}_{ij}$.

	$\mathbb{A}^{\mathtt{max}}$	$\mathbb{L}^{\mathtt{max}}$	$\mathbb{D}^{\mathtt{max}}$
**Calothrix membranacea**	0.121232	0.0177594	0.832519
**Coelastrella rubescens**	0.201587	0.0485413	0.944577
**Fischerella ambigua**	0.144784	0.0128154	0.877442
**Microchaete diplosiphon**	0.308973	0.0233631	0.937951
**Microcoleus autumnalis**	0.050446	0.0158423	0.863109
**Nodularia sphaerocarpa**	0.158121	0.016761	0.86643
**Nostoc commune**	0.559274	0.131888	0.910803
**Plectonema sp.**	0.503704	0.0656241	0.914072

S3 Table
