# Supplementary material for: Weathering of a Roman Mosaic—A Biological and Quantitative Study on In Vitro Colonization of Calcareous Tesserae by Phototrophic Microorganisms
Source: PLoS One. 2016 Oct 26;11(10):e0164487. doi: 10.1371/journal.pone.0164487 (PMC5082677; doi:10.1371/journal.pone.0164487)
Supplement: S4 Table — Average operator of aggregation for the quantities Aij,Lij,Dij. (PDF) [file pone.0164487.s010.pdf]

S4 Table

Average operator. Average operator of aggregation for the quantities $\mathcal{A}_{ij}, \mathcal{L}_{ij}, \mathcal{D}_{ij}$.

	$\overline{\mathbb{A}}$	$\overline{\mathbb{L}}$	$\overline{\mathbb{D}}$
**Calothrix membranacea**	0.055614	0.0125717	0.71311
**Coelastrella rubescens**	0.0848199	0.0152536	0.813758
**Fischerella ambigua**	0.0524913	0.00940145	0.770148
**Microchaete diplosiphon**	0.124394	0.0131955	0.741102
**Microcoleus autumnalis**	0.026233	0.00902597	0.794575
**Nodularia sphaerocarpa**	0.0584345	0.0127763	0.754725
**Nostoc commune**	0.279119	0.0598953	0.829267
**Plectonema sp.**	0.238026	0.0328269	0.829008

S4 Table
