# Supplementary material for: Weathering of a Roman Mosaic—A Biological and Quantitative Study on In Vitro Colonization of Calcareous Tesserae by Phototrophic Microorganisms
Source: PLoS One. 2016 Oct 26;11(10):e0164487. doi: 10.1371/journal.pone.0164487 (PMC5082677; doi:10.1371/journal.pone.0164487)
Supplement: S5 Table — Weighted average operator of aggregation for the quantities Aij,Lij,Dij. (PDF) [file pone.0164487.s011.pdf]

S5 Table

Weighted average operator. Weighted average operator of aggregation for the quantities $\mathcal{A}_{ij}, \mathcal{L}_{ij}, \mathcal{D}_{ij}$.

	$\mathbb{A}^{w}$	$\mathbb{L}^{w}$	$\mathbb{D}^{w}$
**Calothrix membranacea**	0.0695556	0.0134337	0.743628
**Coelastrella rubescens**	0.106201	0.0151554	0.83739
**Fischerella ambigua**	0.0684545	0.0101733	0.802069
**Microchaete diplosiphon**	0.164956	0.0151146	0.802097
**Microcoleus autumnalis**	0.0314827	0.0098008	0.807165
**Nodularia sphaerocarpa**	0.0753755	0.0132472	0.776323
**Nostoc commune**	0.338004	0.0658317	0.849285
**Plectonema sp.**	0.301286	0.0390317	0.857287

S5 Table
