# Supplementary material for: Weathering of a Roman Mosaic—A Biological and Quantitative Study on In Vitro Colonization of Calcareous Tesserae by Phototrophic Microorganisms
Source: PLoS One. 2016 Oct 26;11(10):e0164487. doi: 10.1371/journal.pone.0164487 (PMC5082677; doi:10.1371/journal.pone.0164487)
Supplement: S6 Table — (PDF) [file pone.0164487.s012.pdf]

S6 Table

Rescaled values of quantities $\mathbb{A}_i^{\max}, \mathbb{L}_i^{\max}, \mathbb{D}_i^{\max}$.

	$\mathbb{A}_N^{MAX}$	$\mathbb{L}_N^{MAX}$	$\mathbb{D}^{MAX}$
**Calothrix membranacea**	0.216767	0.134655	0.881368
**Coelastrella rubescens**	0.360444	0.36805	1.
**Fischerella ambigua**	0.258878	0.0971687	0.928926
**Microchaete diplosiphon**	0.552454	0.177144	0.992986
**Microcoleus autumnalis**	0.090199	0.12012	0.913752
**Nodularia sphaerocarpa**	0.282726	0.127085	0.917267
**Nostoc commune**	1.	1.	0.964244
**Plectonema sp.**	0.900639	0.497575	0.967705

S6 Table
