# Supplementary material for: Weathering of a Roman Mosaic—A Biological and Quantitative Study on In Vitro Colonization of Calcareous Tesserae by Phototrophic Microorganisms
Source: PLoS One. 2016 Oct 26;11(10):e0164487. doi: 10.1371/journal.pone.0164487 (PMC5082677; doi:10.1371/journal.pone.0164487)
Supplement: S7 Table — (PDF) [file pone.0164487.s013.pdf]

S7 Table

Rescaled values of quantities $\bar{\mathbb{A}}_{\mathbb{N}i}, \bar{\mathbb{L}}_{\mathbb{N}i}, \bar{\mathbb{D}}_{\mathbb{N}i}$.

	$\overline{\mathbb{A}}_{\mathtt{N}}$	$\overline{\mathbb{L}}_{\mathtt{N}}$	$\overline{\mathbb{D}}$
**Calothrix membranacea**	0.199248	0.209895	0.859927
**Coelastrella rubescens**	0.303885	0.25467	0.981298
**Fischerella ambigua**	0.188061	0.156965	0.928709
**Microchaete diplosiphon**	0.445668	0.220309	0.893683
**Microcoleus autumnalis**	0.0939851	0.150696	0.958165
**Nodularia sphaerocarpa**	0.209353	0.213311	0.910111
**Nostoc commune**	1.	1.	1.
**Plectonema sp.**	0.852775	0.548071	0.999688

S7 Table
