# Supplementary material for: Weathering of a Roman Mosaic—A Biological and Quantitative Study on In Vitro Colonization of Calcareous Tesserae by Phototrophic Microorganisms
Source: PLoS One. 2016 Oct 26;11(10):e0164487. doi: 10.1371/journal.pone.0164487 (PMC5082677; doi:10.1371/journal.pone.0164487)
Supplement: S8 Table — (PDF) [file pone.0164487.s014.pdf]

S8 Table

Rescaled values of quantities $\mathbb{A}_i^w, \mathbb{L}_i^w, \mathbb{D}_i^w$.

	$\mathbb{A}_N{}^w$	$\mathbb{L}_N{}^w$	$\mathbb{D}^w$
**Calothrix membranacea**	0.205783	0.204061	0.867419
**Coelastrella rubescens**	0.3142	0.230214	0.976791
**Fischerella ambigua**	0.202526	0.154536	0.93559
**Microchaete diplosiphon**	0.488029	0.229595	0.935622
**Microcoleus autumnalis**	0.093143	0.148877	0.941534
**Nodularia sphaerocarpa**	0.223002	0.201229	0.905558
**Nostoc commune**	1.	1.	0.990666
**Plectonema sp.**	0.89137	0.592901	1.

S8 Table
